# The aberrant upstream pathway regulations of CDK1 protein were implicated in the proliferation and apoptosis of ovarian cancer cells

**DOI:** 10.1186/s13048-017-0356-x

**Published:** 2017-09-12

**Authors:** Ruitao Zhang, Huirong Shi, Fang Ren, Minghui Zhang, Pengcheng Ji, Wenwen Wang, Chuanna Liu

**Affiliations:** Department of Gynecology, First Affiliated Hospital, Zhengzhou University, NO.1 Jianshe Road, Zhengzhou, 450052 Henan People’s Republic of China

**Keywords:** Cyclin dependent kinases 1, G2/M phase regulation, Ovarian epithelial cancer, Proliferation, Apoptosis

## Abstract

**Background:**

Upregulation of Cyclin dependent kinase 1 (CDK1) protein is closely related with the prognosis of several malignant tumors. Chk1-CDC25C-CDK1 signaling and P53-P21WAF1-CDK1 signaling pathways are closely related with the cell cycle G2/M phase regulation. The present study aimed to analyze the relationship between CDK1 and the proliferation and apoptosis of ovarian cancer cells, investigate its molecular mechanism preliminarily.

**Methods:**

The specific short-hair RNA (shRNA) plasmids and negative control plasmid of CDK1, checkpoint kinase 1 (CHK1) and p53 genes were transfected into ovarian cancer SK-OV-3 and OVCAR-3 cells respectively. The expressions of CDK1, CHK1 and p53 mRNA and CDK1, Chk1 and P53 protein were detected by sqRT-PCR and Western blot, levels of phospho-CDK1(Thr14/Tyr15), CyclinB1, phospho-Chk1(ser345), cell division cycle 25C (CDC25C), phospho-CDC25C(ser216), P21WAF1, phospho-P53(ser15), proliferating cell nuclear antigen (PCNA), Ki-67, Bcl-2, Bax, Caspase8, Cleaved-caspase3 and Cytochrome C were examined by Western blot. The cell proliferation was measured by MTT and Trypan blue exclusion assay respectively, the cell cycle phase distribution and cell apoptosis rate were detected by flow cytometry (FCM) assay.

**Results:**

As results of CDK1 inhibition by shRNA, the cell proliferation was repressed, the cell numbers of G2/M phase and cell apoptosis rate were increased in both SK-OV-3 and OVCAR-3 cells. After knockdown of CDK1, expressions of PCNA, Ki-67 and Bcl-2 protein were downregulated, expressions of Bax, Caspase8, Cleaved-caspase3 and Cytochrome C were upregulated. While knockdown the CHK1 and p53 by shRNA respectively, the similar effects were observed on the cell proliferation, cell cycle phase distribution and apoptosis in both SK-OV-3 and OVCAR-3 cells, as well as the expressions of the proliferation and apoptosis related proteins mentioned above. Moreover, the levels of p-CDK1(Thr14/Tyr15) were increased after either CHK1 inhibition or p53 inhibition.

**Conclusions:**

Abnormal activation of CDK1 was implicated in the proliferation and apoptosis regulation of ovarian cancer cells, which might be due to the aberrant regulations of the upstream Chk1-CDC25C and P53-P21WAF1 signaling pathway.

## Background

Ovarian cancer leads to the most unfavorable prognosis and highest mortality rates in female genital system malignant tumors, which is compared to cervical cancer and endometrial carcinoma [1]. Ovarian cancer often was characterized by insidious onset, diagnosis at late stage, dissemination, relapse, and tendency to develop chemotherapy resistance. Because of the unclear etiology, early diagnosis and treatment cannot be implemented effectively in ovarian cancer patients. Therefore, the efforts aim to elucidate the molecular mechanism of ovarian carcinoma may be helpful for the diagnosis and therapy of ovarian cancer, even improving the prognosis and reducing mortality finally.

The development and progression of human cancer is a multistep and complex process, which is verified to be closely related with the misregulation of cell cycle and aberrant cell signaling pathway transduction [2]. G2/M phase arrest is the most crucial cell cycle protective barrier for cell DNA damage repair before cell enters mitosis [3]. CDK1 protein is the core factor and plays key roles in the cell cycle G2/M phase regulation network [4]. It had been demonstrated that upregulated expressions of CDK1 protein were detected in many human malignant tumor tissues, including laryngeal cancer, esophageal cancer, lung cancer, hepatocellular carcinoma, colorectal cancer, kidney cancer and ovarian cancer, which was closely related to the malignant prognosis [5–11]. Our previous study has shown upregulated expressions of CDK1, P53, and downregulated expression of P21WAF1 were detected in epithelial ovarian cancer tissues, which indicated abnormal expressions of CDK1, P53 and P21WAF1 were related to the tumorigenesis of ovarian cancer [12].

In present study, specific short-hair RNA plasmids of CDK1 was used to knockdown the expression of CDK1 in the human ovarian cancer SK-OV-3 and OVCAR-3 cells respectively. Effects of the CDK1 inhibition to the cell proliferation, cell cycle phase distribution and apoptosis rate were measured. Furthermore, expressions of CHK1 and p53 were inhibited by shRNA plasmids transfection respectively, which aimed to analyze the possible molecular mechanism of CDK1 functions in proliferation and apoptosis of ovarian cancer.

## Materials and methods

### Cell transfection

Human ovarian carcinoma SK-OV-3 and OVCAR-3 cells were purchased from Chinese Academy of Sciences Cell Bank (Shanghai, China), and cultured in completed RPMI-1640 medium (HyClone, Logan, Utah, USA), at 37 °C with 5% CO_2_. Cells were harvested in logarithmic phase of growth for all experiments described below. CDK1, CHK1 and p53 shRNA lentiviral plasmids and negative control plasmid (Santa Cruz Biotechnology, Santa Cruz, Calif., USA) were used for cell transfection respectively, which was performed following the protocol of shRNA Plasmid Transfection Reagent (Santa Cruz Biotechnology, Santa Cruz, Calif., USA). Stably transfected SK-OV-3 and OVCAR-3 cells were isolated by puromycin (Clontech, Calif., USA) selection after tansfection 48 h. Three cell groups used for next step work were blank control cells (B), negative control shRNA cells (NC) and knockdown shRNA cells (K).

### sqRT-PCR

Cell total RNA was isolated using Trizol Reagent (Invitrogen, Carlsbad, Calif., USA), and first strand cDNA was synthesized from 1 μg total RNA according to the protocol of RevertAid first strand cDNA synthesis kit (Fermentas, EU). Primers used in sqRT-PCR were CDK1, CHK1 and p53 (Santa Cruz Biotechnology, Santa Cruz, Calif., USA), and β-actin 5′-ACGCACCCCAACTACAACTC-3′ (sense) and 5′-TCTCCTTAATGTCACGCACGA-3′ (antisense). PCR cycling parameters (19 cycles) were: denaturation (94 °C,30s), annealing (56 °C,30s) and extension (72 °C,30s). Equal amounts of PCR products were electrophoresed on 1.2% agarose gels and visualized by ethidium bromide staining. The specific bands of PCR products were analyzed by Image-Pro Plus 6.0 system, β-actin was used as a control for normalization. RT-PCR was performed for three times independently.

### Western blot

The antibodies used in the Western blot, following manufacturer’s protocols, were mouse anti-human monoclonal CDK1, rabbit anti-human polyclonal phospho-CDK1(Thr14/Tyr15), mouse anti-human monoclonal CyclinB1, mouse anti-human monoclonal Chk1, goat anti-human polyclonal phospho-Chk1(ser345), mouse anti-human monoclonal CDC25C, goat anti-human polyclonal phospho-CDC25C(ser216), goat anti-human polyclonal Ki-67, mouse anti-human monoclonal Cytochrome C (Santa Cruz Biotechnology, Santa Cruz, Calif., USA), mouse anti-human monoclonal P53, rabbit anti-human polyclonal phospho-P53 (Ser15), mouse anti-human monoclonal P21_WAF1_, mouse anti-human monoclonal PCNA, rabbit anti-human polyclonal Bcl-2, mouse anti-human monoclonal Bax, rabbit anti-human polyclonal Caspase8, mouse anti-human monoclonal Cleaved-caspase3 and mouse anti-human monoclonal β-actin (Beyotime Biotechnology, Haimen, Jiangsu, China). Total protein was extracted using RIPA Lysis Buffer for Western and IP (Beyotime Biotechnology, Haimen, Jiangsu, China), and protein concentration was determined using BCA assay. Equal amounts of protein (30 μg) were separated by 10% SDS-PAGE and transferred onto PVDF membranes. The detection of hybridized protein was performed by enhanced chemiluminescence kit (Zhongshan Goldenbridge Biotechnology, Peking, China), β-actin was used as a control for normalization. The relative values of specific bands were analyzed by Image-Pro Plus 6.0 system.

### MTT proliferation assay

Planted 1 × 10^4^ cells per well into 96-well plates, and added 100 μl medium containing 10% FBS into each well. Five duplicate wells were set up for each group. Cultured cells continuously for 5 days, added 20 μl MTT reagent (5 mg/mL, Sigma, St. Louis, USA) into each well, incubated for another 4 h then aspirated former medium and added 150 μl DMSO. The absorbance of sample was measured by Microplate spectrophotometer (Thermo, Spectronic, Madison, WI, USA) at 492 nm. All experiments were done in triplicate. Cell growth curve was plotted versus time by origin 8 software.

### Trypan blue exclusion assay

Trypan blue exclusion assay was performed following the protocol of Trypan Blue Staining Cell Viability Assay Kit (Beyotime Biotechnology, Haimen, Jiangsu, China). Mixed 100 μl single cell suspension solution with 100 μl trypan blue solution. After 3 min, this mixture was evaluated under a light microscope (100 times magnification) using hemacytometer plate where blue-colored cells were considered nonviable. The ratio of unstained cell numbers to total cell numbers was reported as the viability percentage for each cell category.

### Flow cytometry cell cycle distribution analysis

About 1 × 10^6^ cells were treated into single cell suspension with PBS solution for twice, fixed by 70% ice ethanol for 24 h, added propidium iodide solution (containing 100 mg/L RNaseA) after PBS washing and centrifugation, incubated at room temperature away from light. Then, cell cycle distribution was measured with FACScan system (BD Biosciences, San Jose, CA, USA), and analyzed by Mulpicycle for Windows software.

### Flow cytometry cell apoptosis analysis

About 1 × 10^6^ cells were treated into single cell suspension with PBS solution, and were prepared following manufacture’s protocol of Annexin V-FITC Apoptosis Detection Kit (Beyotime Biotechnology, Haimen, Jiangsu, China). Then, rates of apoptosis were analyzed with FACScan system (BD Biosciences, San Jose, CA, USA).

### Statistical analysis

Average values were expressed as mean ± standard deviation (SD). Measurement data were analyzed by one-way ANOVA and Bonferroni test using SPSS 17.0 software package. Difference was considered significant when *P* value was less than 0.05.

## Results

### Effects of CDK1 knockdown on the SK-OV-3 and OVCAR-3 cells

**Fig. 1:**
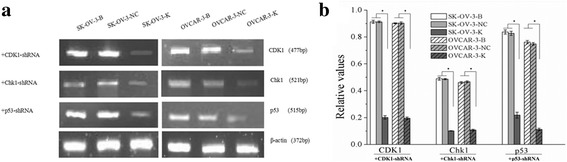
Expressions of CDK1, CHK1 and p53 mRNA in each ovarian cancer cells groups measured by sqRT-PCR (B: Blank, NC: Negative Control, K: Knockdown). Expressions of CDK1, CHK1 and p53 mRNA in ovarian cancer cells were downregulated after CDK1, CHK1 and p53 RNAi respectively. Histogram graphs show relative values of each group cells measured by sqRT-PCR. Each bar represents the mean ± SD.^*^ *P* < 0.05

**Fig. 2:**
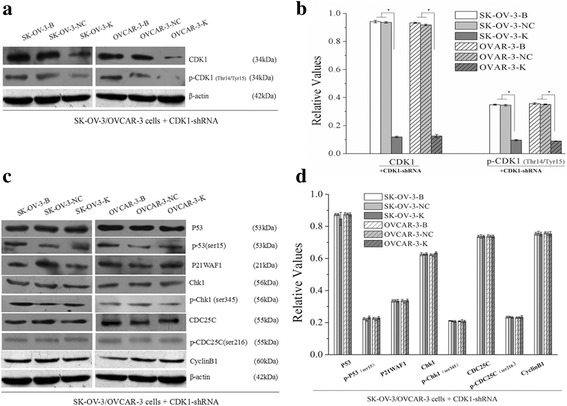
Effects of CDK1 knockdown on the expressions of G2/M checkpoint related signaling proteins in ovarian cancer cells measured by western blot (B: Blank, NC: Negative Control, K: Knockdown). Knockdown of CDK1 in ovarian cancer cells could decrease the expressions of CDK1 and p-CDK1 proteins, but had no effects on its upstream signaling proteins. Histogram graphs show relative values of each group cells measured by western blot. Each bar represents the mean ± SD.^*^ *P* < 0.05

After CDK1 knockdown, expressions of CDK1 mRNA and CDK1 protein, and p-CDK1(Thr14/Tyr15) were downregulated in SK-OV-3-K and OVCAR-3-K cells (Fig. 1, Fig. 2a, b). Moreover, expressions of P53, p-P53(ser15), P21WAF1, Chk1, p-Chk1(ser345), CDC25C, p-CDC25C(ser216) and CyclinB1 proteins had no significant differences after CDK1 silencing in SK-OV-3-K and OVCAR-3-K cells (Fig. 2c, d).

**Fig. 3:**
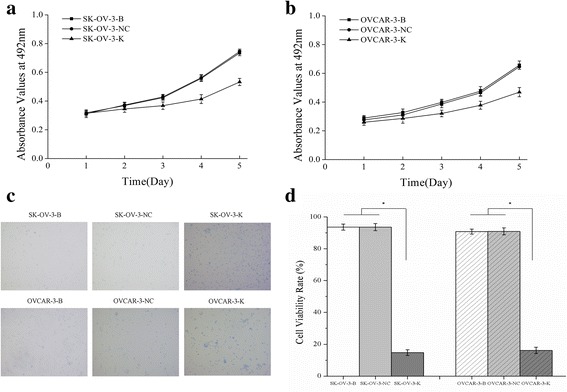
Effects of CDK1 knockdown on the proliferation of ovarian cancer cells measured by MTT assay and Trypan blue exclusion assay. **a, b** Knockdown of CDK1 could inhibit the proliferation of ovarian cancer cells measured by MTT assay. **c** Knockdown of CDK1 could inhibit the viability of ovarian cancer cells detected by Trypan blue exclusion assay (Trypan blue staining, ×100). **d** Histogram graphs show the cell viability rate of ovarian cancer cells detected by Trypan blue exclusion assay after CDK1 silencing. Each bar represents the mean ± SD

**Fig. 4:**
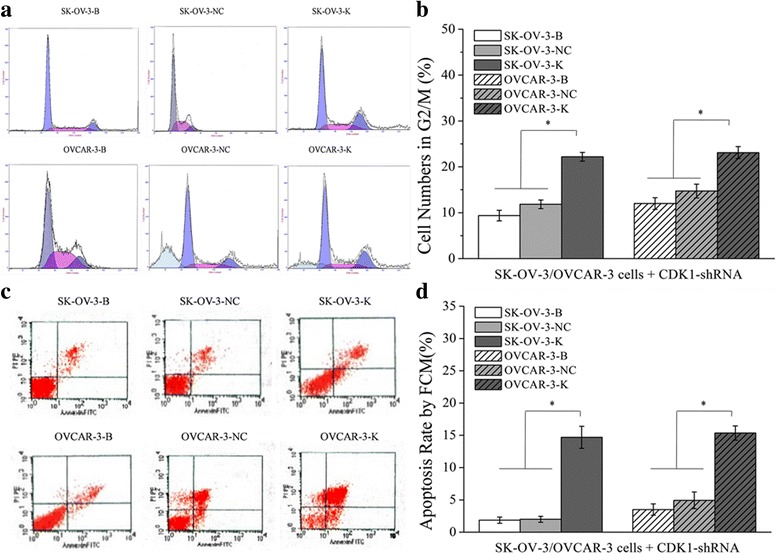
Effects of CDK1 knockdown on cell numbers in G2/M phase and cell apoptosis rate detected by FCM assay. Knockdown of CDK1 in ovarian cancer cells could increase the cell numbers of G2/M phase and the cell apoptosis rate. **a, b** Differences of cell numbers in G2/M phase of each cell groups detected by flow cytometry assay **(**FCM). **c, d** Differences of cell apoptosis rate of each cell groups detected by FCM**.** Histogram graphs show differences of cell numbers in G2/M phase and cell apoptosis rate in each cell groups. Each bar represents the mean ± SD.^*^ *P* < 0.05

As a result of CDK1 inhibition, cell proliferation was obviously repressed as detected by MTT (Fig. 3a,b) and trypan blue exclusion assay (Fig. 3c,d). Cell numbers in G2/M phase and cell apoptosis rate were significantly increased (Fig. 4) in both SK-OV-3-K and OVCAR-3-K cells which were measured by flow cytometry assay.

**Fig. 5:**
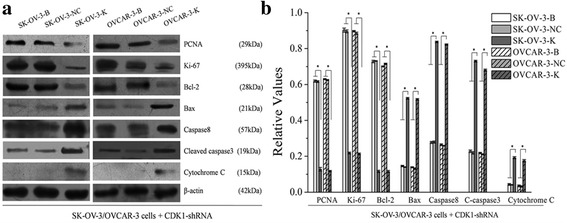
Effects of CDK1 knockdown on the expressions of proliferation and apoptosis related proteins of ovarian cancer cells measured by western blot (B: Blank, NC: Negative Control, K: Knockdown). Knockdown of CDK1 in ovarian cancer cells could affect the expressions of its downstream proliferation and apoptosis related proteins. Histogram graphs show relative values of each group cells measured by western blot. Each bar represents the mean ± SD.^*^ *P* < 0.05

Expressions of proliferation and apoptosis related proteins were also measured in each cell groups. After CDK1 silencing, downregulations of PCNA, Ki-67 and Bcl-2 proteins, and upregulations of Bax, Caspase8, Cleaved-caspase3 and Cytochrome C proteins were observed both in SK-OV-3-K and OVCAR-3-K cells, no differences were observed between blank and NC group cells (Fig. 5).

### Effects of CHK1 knockdown on the SK-OV-3 and OVCAR-3 cells

**Fig. 6:**
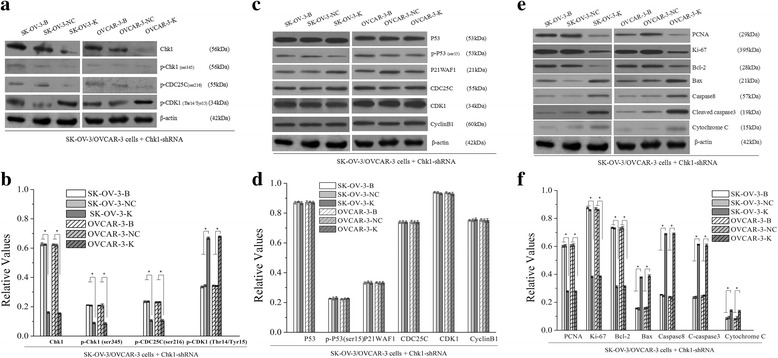
Effects of CHK1 knockdown on the expressions of G2/M related signaling proteins and proliferation and apoptosis related proteins measured by western blot (B: Blank, NC: Negative Control, K: Knockdown). Knockdown of CHK1 in ovarian cancer cells could decrease the activities of Chk1-CDK1 signaling pathway, and affect the expressions of its downstream proliferation and apoptosis related proteins. Histogram graphs show relative values of each group cells measured by western blot. Each bar represents the mean ± SD.^*^ *P* < 0.05

**Fig. 7:**
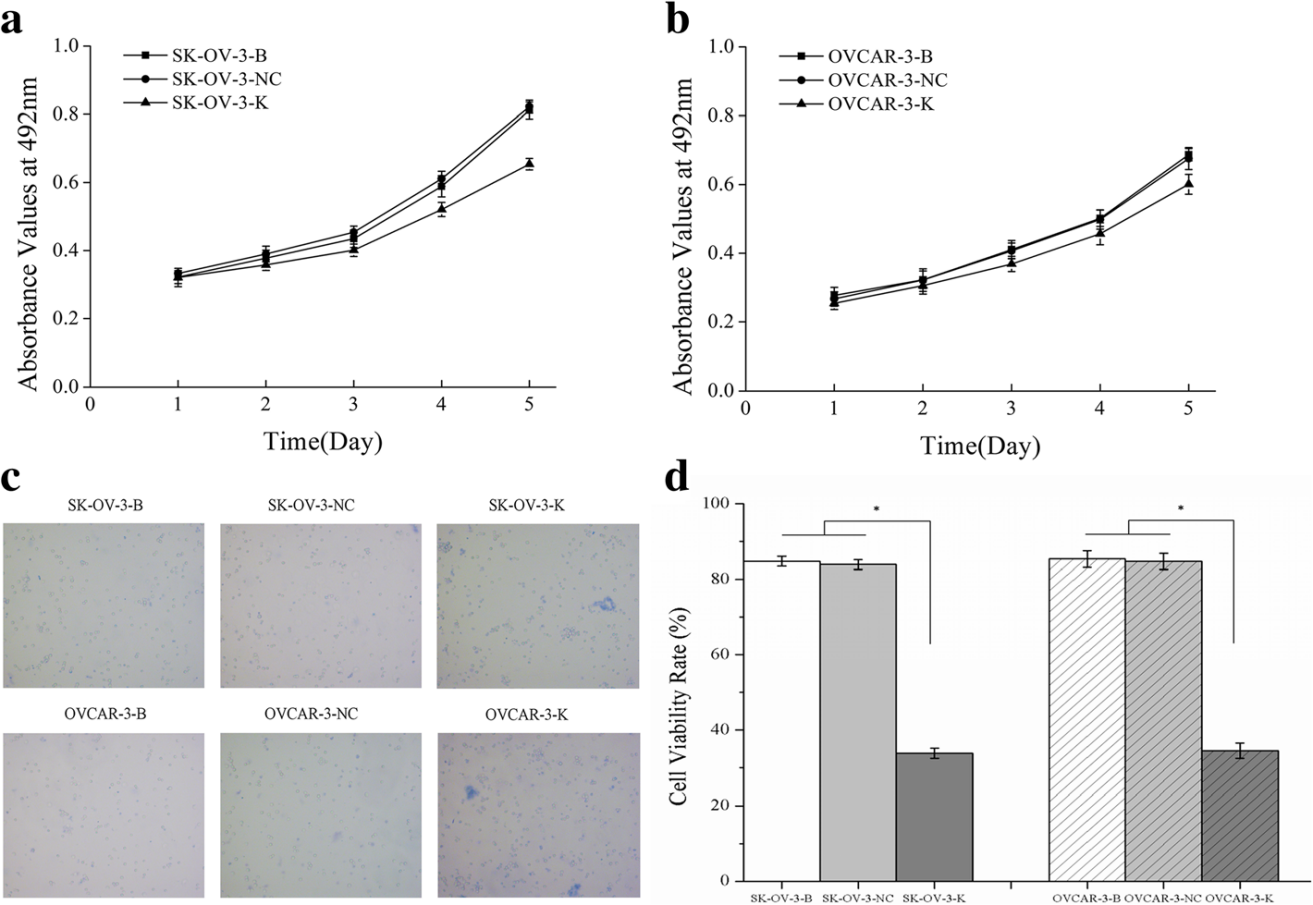
Effects of CHK1 knockdown on the proliferation of ovarian cancer cells measured by MTT assay and Trypan blue exclusion assay. **a**, **b** Knockdown of CHK1 could inhibit the proliferation of ovarian cancer cells measured by MTT assay. **c** Knockdown of CHK1 could inhibit the viability of ovarian cancer cells detected by Trypan blue exclusion assay (Trypan blue staining, ×100). **d** Histogram graphs show the cell viability rate of ovarian cancer cells detected by Trypan blue exclusion assay after CHK1 silencing. Each bar represents the mean ± SD

**Fig. 8:**
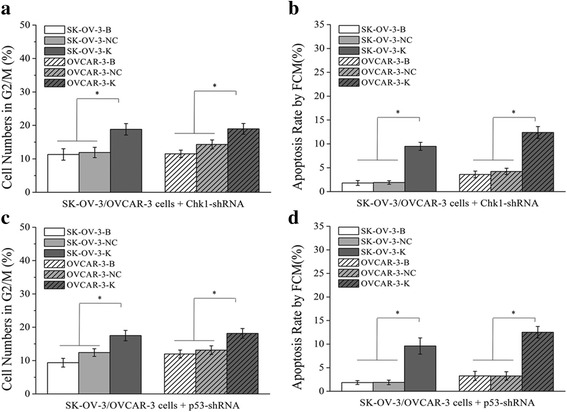
Effects of CHK1 or p53 knockdown on cell numbers in G2/M phase and cell apoptosis detected by FCM assay. Knockdown of CHK1 or p53 in ovarian cancer cells could increase the cell numbers of G2/M phase and the cell apoptosis rate. **a**, **b** Effects of CHK1 knockdown on cell numbers in G2/M phase and cell apoptosis rate of ovarian cancer cells. **c**, **d** Effects of p53 knockdown on cell numbers in G2/M phase and cell apoptosis rate of ovarian cancer cells. Each bar in Histogram graphs represents the mean ± SD.^*^ *P* < 0.05

After CHK1 knockdown, expressions of CHK1 mRNA and Chk1 protein were all downregulated in SK-OV-3-K and OVCAR-3-K cells (Fig. 1, Fig. 6a, b). Repressed cell proliferation (Fig. 7), increased cell numbers in G2/M phase and cell apoptosis rate (Fig. 8a, b) were observed in both SK-OV-3-K and OVCAR-3-K cells while CHK1 was inhibited by shRNA plasmid. However, all the difference folds were less than those following CDK1 knockdown.

Expressions of P53, p-P53(ser15), P21WAF1, CDC25C, CDK1 and CyclinB1 proteins had no significant differences after CHK1 silencing, whereas obvious downregulations of p-Chk1(ser345) and p-CDC25C(ser216) proteins, and significant upregulation of p-CDK1(Thr14/Tyr15) protein were observed in SK-OV-3-K and OVCAR-3-K cells respectively (Fig. 6a, b, c, d).

Regulation changes of proliferation and apoptosis related proteins mentioned above were observed both in SK-OV-3-K and OVCAR-3-K cells after CHK1 knockdown which were similar to CDK1 knockdown. However, all the difference folds were less than those following CDK1 knockdown (Fig. 6e, f).

### Effects of p53 knockdown on the SK-OV-3 and OVCAR-3 cells

**Fig. 9:**
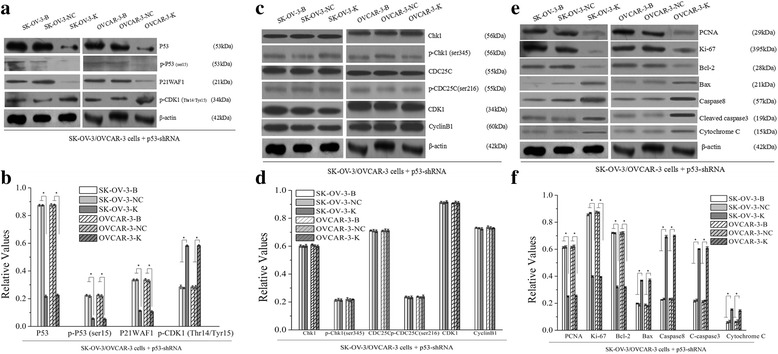
Effects of p53 knockdown on the expressions of G2/M related signaling proteins and proliferation and apoptosis related proteins measured by western blot (B: Blank, NC: Negative Control, K: Knockdown). Knockdown of p53 in ovarian cancer cells could decrease the activities of p53-CDK1 signaling pathway, and affect the expressions of its downstream proliferation and apoptosis related proteins. Histogram graphs show relative values of protein expression in each group cells measured by western blot. Each bar represents the mean ± SD.^*^ *P* < 0.05

**Fig. 10:**
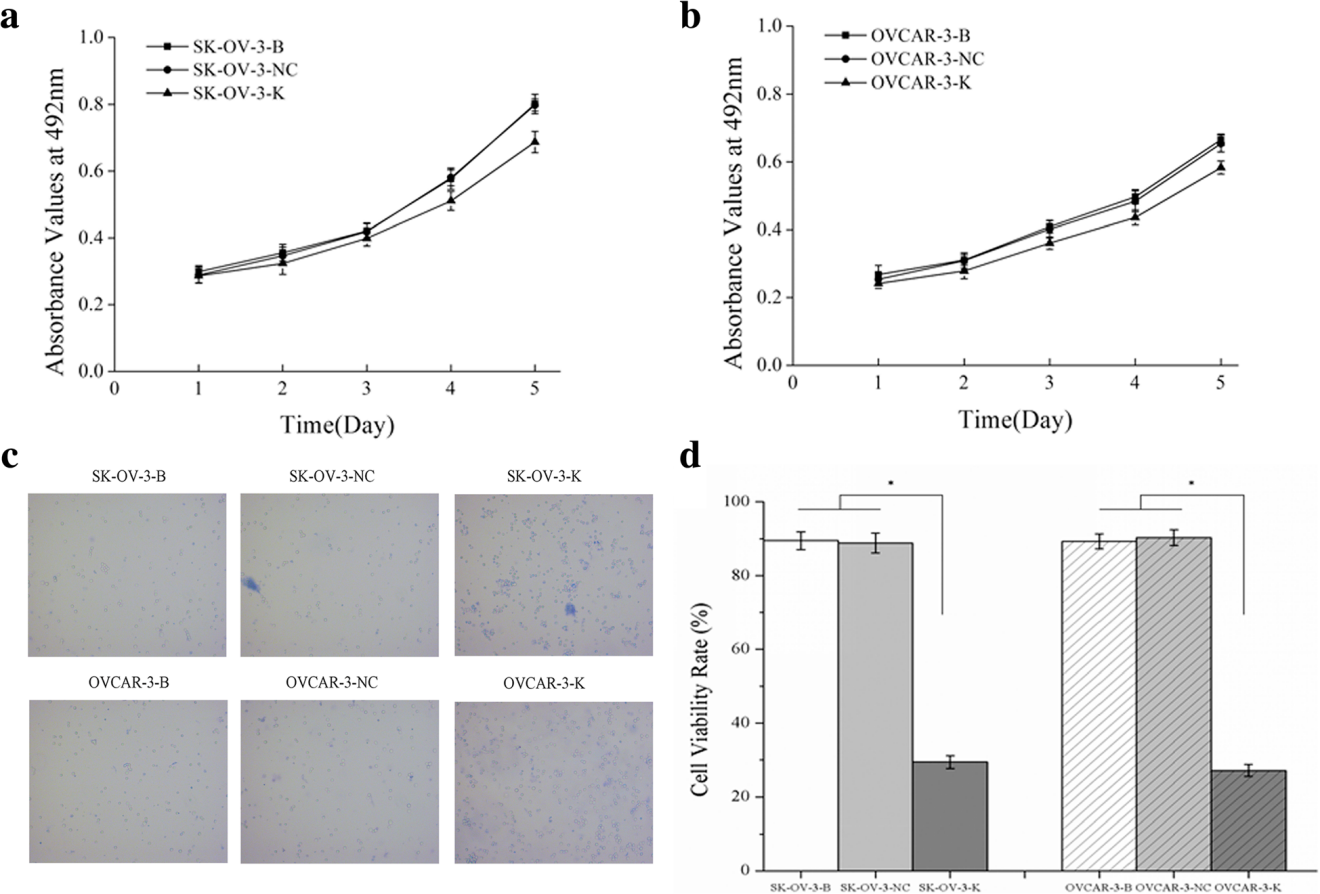
Effects of p53 knockdown on the proliferation of ovarian cancer cells measured by MTT assay and Trypan blue exclusion assay. **a, b** Knockdown of p53 could inhibit the proliferation of ovarian cancer cells measured by MTT assay. **c** Knockdown of p53 could inhibit the viability of ovarian cancer cells detected by Trypan blue exclusion assay (Trypan blue staining, ×100). (**d)**Histogram graphs show the cell viability rate of ovarian cancer cells detected by Trypan blue exclusion assay after p53 silencing. Each bar represents the mean ± SD

After p53 knockdown, expressions of p53 mRNA and P53 protein were all downregulated in SK-OV-3-K and OVCAR-3-K cells (Fig. 1, Fig. 9a, b). Similar results that repressed cell proliferation (Fig. 10), increased cell numbers in G2/M phase and cell apoptosis rate (Fig. 8c, d) were observed in both SK-OV-3-K and OVCAR-3-K cells when p53 was inhibited by shRNA plasmid. Likewise, all the difference folds were less than those following CDK1 knockdown.

Expressions of Chk1, p-Chk1(ser345), CDC25C, p-CDC25C(ser216), CDK1 and CyclinB1 proteins had no significant differences after p53 silence, whereas obvious downregulations of p-P53(ser15) and P21WAF1 proteins, and significant upregulation of p-CDK1(Thr14/Tyr15) protein were observed in SK-OV-3-K and OVCAR-3-K cells respectively (Fig. 9a, b, c, d).

Regulation changes of proliferation and apoptosis related proteins mentioned above were observed both in SK-OV-3-K and OVCAR-3-K cells after p53 knockdown which were similar to CDK1 knockdown. Similarly, all the difference folds were less than those following CDK1 knockdown (Fig. 9e, f).

## Discussion

Cell DNA is continuously damaged by exogenous and endogenous factors in the cell cycle running process, cell cycle could be arrested by activation of DNA damage checkpoints when cell DNA damage was detected [2]. DNA damage checkpoints function to perform cell DNA repair before entering mitosis or induce apoptosis, otherwise the damaged genetic materials accumulation may lead to cells become cancerous finally [13]. The main DNA damage checkpoints in cell cycle include G1/S phase checkpoint, S phase checkpoint and G2/M phase checkpoint, and G2/M phase checkpoint is the foremost one and the last cell DNA repair protective barrier which determines whether cell proceed mitosis or apoptosis [3, 14].

CDK1/cyclinB1 compound, also called maturation promoting factor (MPF), is the indispensable protein kinase for cell cycle G2/M phase transformation in eukaryotic mitosis. Mitosis could only be triggered by activation of MPF [15]. CDK1 protein contains Thr161 amino acid activation site and Thr14/Tyr15 amino acid inhibition sites. CDK1 protein could be activated by phosphorylation of Thr161 site and dephosphorylation of Thr14/Tyr15 sites. However, the phosphorylation or dephosphorylation status of Thr14/Tyr15 sites is pivotal for the activation of CDK1, which directly determines the activity of MPF [16, 17].

Upregualtion of CDK1 protein was also reported to be relevant to the development and progress of ovarian cancer. Overexpression of CDK1 was detected in ovarian cancer tissues, which was related with the worse prognosis, and could be a potential molecular biomarkers of epithelial of ovarian cancer [18, 19]. Because of high expression of CDK1 in ovarian cancer tissue, there was study suggested that aberrant expression of CDK1 could be an early event of ovarian cancer [20]. In our previous study, high levels CDK1 were detected in almost all ovarian cancer tissues, which were not related with the clinical stage and histological differentiation of ovarian cancer [12]. Here, our results showed that cell proliferation was restrained, cell numbers of G2/M phase and cell apoptosis rate were increased when expression of CDK1 was silenced by RNAi in SK-OV-3 and OVCAR-3 cells. Furthermore, downregulations of PCNA, Ki-67 and Bcl-2 proteins, and upregulations of Bax, Caspase8, Cleaved-caspase3 and Cytochrome C proteins were observed both in SK-OV-3 and OVCAR-3 cells after CDK1 silencing. These data indicated that upregulation of CDK1 and aberrant activation of CDK1 was implicated in the regulation of cell proliferation and apoptosis in ovarian cancer cells. Hence, we speculated that, aberrant activation of CDK1 might enhance the activity of MPF and promote ovarian cancer cells mitosis and proliferation persistently.

In G2/M phase DNA damage checkpoint, there are two signaling transduction pathways performing DNA damage signaling transmission, called CDC25C signaling and P53 signaling for short. Cell DNA damage can activate G2/M checkpoint and induce CDC25C signaling and P53 signaling transduction to restrain the activation of CDK1/cyclinB1 compound, and restrict MPF access into the cell nucleus, finally causes cell cycle arrested at G2/M phase to repair DNA damage or induce cell apoptosis [21].

As downstream transduction protein, Chk1 can be activated with phosphorylation of Ser345 site by receiving cell DNA damage signaling from ATM/ATR [22, 23]. Activated Chk1 induces CDC25C to be phosphorylated at Ser216 site and to be combined with 14–3-3σ, which makes CDC25C deactivated, finally leads inhibition of MPF activity and G2/M phase arrest to repair DNA damage or induce cell apoptosis [24]. Deactivated CDC25C can be reactivated with dephosphorylation of Ser216 site by protein phosphatase 1 (PP1) [25].

More than 50% human cancers contain p53 gene mutations and mutant P53 protein expression [26]. However, wild-type p53 plays a key role in the regulatory of cell cycle, programmed cell death, and cell differentiation [3]. In response to DNA damage and cell stress signals, P53 can be activated directly with phosphorylation of Ser15 site or indirectly by ATM/ATR with phosphorylation of Ser15 site in G2/M checkpoint [27, 28]. P21WAF1 functions as a main trancriptional target of p53. In G2/M checkpoint, activated P53 induces P21WAF1 to bind to and inhibit the activity of CDK1, finally causes cell cycle arrest to repair DNA damage or induce cell apoptosis [21, 29].

Intended to analyze the possible mechanism of CDK1 functions on regulation of cell proliferation and apoptosis in ovarian cancer cells, several upstream regulator proteins in CDC25C and P53 signaling pathways were measured. As a result of CDK1 inhibition, CDK1 and p-CDK1(Thr14/Tyr15) proteins were all downregulated in SK-OV-3 and OVCAR-3 cells. However, expressions of regulator proteins, including P53, p-P53(ser15), P21WAF1, Chk1, p-Chk1(ser345), CDC25C p-CDC25C(ser216) and CyclinB1 were unaffected by CDK1 silencing. And then, expressions of CHK1 and p53 gene were knockdown by RNAi in SK-OV-3 and OVCAR-3 cells respectively. After CHK1 or p53 inhibition, repression of cell proliferation, increase of apoptosis and cell numbers of G2/M phase, and differences of proliferation and apoptosis related proteins were observed, which were similar to CDK1 silence. Moreover, expressions of Chk1, p-Chk1(ser345) and p-CDC25C(ser216) proteins were downregulated, and expressions of p-CDK1(Thr14/Tyr15) were upregulated in both SK-OV-3 and OVCAR-3 cells after CHK1 knockdown. But, expressions of P53, p-P53(ser15), P21WAF1, CDC25C, CDK1 and CyclinB1 proteins had no significant differences after CHK1 silence. In the other hand, expressions of P53, p-P53(ser15) and P21WAF1 proteins were downregulated, and expressions of p-CDK1(Thr14/Tyr15) were upregulated in both SK-OV-3 and OVCAR-3 cells after p53 knockdown. But, expressions of Chk1, p-Chk1(ser345), CDC25C, p-CDC25C(ser216), CDK1 and CyclinB1 proteins had none significant differences after p53 silencing. In addition, expression differences of proliferation and apoptosis related proteins were also observed in SK-OV-3 and OVCAR-3 cells after inhibition of CHK1 and p53 respectively, which were similar to CDK1 silencing. Together, these data indicated that there was aberrant regulation of Chk1-CDC25C-CDK1/CyclinB1 and P53-P21WAF1-CDK1/CyclinB1 signaling pathways transduction in ovarian cancer cells, and only the activity of CDK1 protein, not the expression of CDK1 protein was regulated by the CDC25C signaling and P53 signaling pathway.

## Conclusions

In general, the present study suggested that abnormal activation of CDK1 was implicated in the proliferation and apoptosis regulation of ovarian cancer cells, which might due to the aberrant regulations of the upstream Chk1-CDC25C and P53-P21WAF1 signaling pathway. Based on present results, further study performed about CDK1 protein functions might be helpful to illuminate the molecular mechanism of the carcinogenesis of ovarian cancer.

## Abbreviations

ATM: ATM serine/threonine kinase
ATR: ATR serine/threonine kinase
CDC25C: Cell division cycle 25C
CDK1: Cyclin dependent kinase 1
Chk1: Checkpoint kinase 1
MPF: Maturation promoting factor
PCNA: Proliferating cell nuclear antigen
PP1: Protein phosphatase 1
RNAi: RNA interference
shRNA: Small hairpin RNA
sqRT-PCR: Semiquantitative reverse transcription polymerase chain reaction
